# Mendelian randomization analyses for the causal relationship between early age at first sexual intercourse, early age at first live birth, and postpartum depression in pregnant women

**DOI:** 10.3389/fpsyt.2024.1287934

**Published:** 2024-04-08

**Authors:** Xuemin Zhao, Linfei Liu

**Affiliations:** ^1^ Department of Internal Medicine, Chengde Medical University, Chengde, China; ^2^ Sericultural Research Institute, Chengde Medical University, Chengde, China

**Keywords:** age at first birth, age at first sexual intercourse, postpartum depression, causal relationship, sex education

## Abstract

**Introduction:**

There are insufficient epidemiological studies on the impact of age at first sexual intercourse (AFS) and age at first live birth (AFB) on postpartum depression (PPD) in pregnant women, and the conclusions of these studies are inconsistent.

**Methods:**

We performed a Mendelian randomization (MR) study to determine the causal relationship between AFS or AFB and the risk of PPD. The summary data were extracted from genome-wide association study (GWAS) summary datasets. We selected the instrumental variables according to the *P* value of exposure-related single nucleotide polymorphisms (*P*<5 ×10^-9^ for AFS and *P*<5 ×10^-8^ for AFB) and estimated the linkage disequilibrium using the clump parameter (10,000 kb, r^2^ < 0.001). Single nucleotide polymorphisms were considered instrumental variables that were significantly associated with exposure factors without linkage disequilibrium. The F-statistics of the instrumental variables should all be larger than 10. A random-effects model of IVW was constructed as the main method in our study.

**Results and discussion:**

MR studies based on GWAS data revealed that both AFS (OR = 0.4, *P <*0.001) and AFB (OR = 0.38, *P <*0.001) were negatively correlated with the risk of PPD. Early AFS and early AFB should be studied as possible risk factors for PPD in the future. Public health departments should attach importance to sex education for young girls. The results of our TSMR should be verified by high-quality prospective epidemiological studies in the future.

## 1 Introduction

Postpartum depression (PPD) negatively impacts approximately 10-30% of mothers, with suicide accounting for approximately 20% of postpartum deaths (1, 2). In addition to having adverse effects on mothers’ health for several months or even one year, PPD may also have serious consequences for children and families (3, 4). PPD is not conducive to forming a healthy mother−child relationship which is important for child development and may affect maternal attachment, sensitivity and parenting style, making children more prone to malnutrition and delaying development (5, 6). PPD patients are at increased risk of developing depressive episodes in the future and are more likely to be diagnosed with bipolar disorder (7, 8).

Identifying the risk factors for PPD is necessary for its effective prevention. To date, various environmental or genetic risk factors that may lead to PPD have been comprehensively studied, but the etiology and pathophysiology of PPD are not fully understood (9). The powerful risk factors for PPD include a history of psychiatric illness, such as depression; gestational diabetes; risky pregnancy; domestic violence; and lack of social support (5, 10, 11).These risk factors for PPD reduce individuals’ ability to cope with crisis conditions and increase susceptibility to stress. Stress and previous adverse life events, which are associated with neuroendocrine changes, are important risk factors for PPD (12). One study showed that there was no significant relationship between PPD and the age of first labor (13). Another study suggested a nonlinear correlation between age at first birth (AFB) and the risk of PPD (14). Adolescent sexual behavior may be harmful to female adolescent health and well-being (15, 16). Therefore, we speculate that early age at first sexual intercourse (AFS) and early age at first birth (AFB) are possible risk factors for PPD.

Both AFS and AFB have an impact on health (17). In Australia, 83% of 3848 surveyed students aged 15-24 years had sexual intercourse; the median age was 16 years, and there was no significant gender difference (18). In Transky, South Africa, teenagers make up approximately 25% of all mothers. However, epidemiological studies investigating the relationship between AFB and PPD, which are sensitive to confounding factors are very limited, and the results are controversial (13, 14). Genotypes are randomly assigned from parents to children, and confounding factors cannot affect the association between genetic variations (GVs) and outcomes, which indicates that their causal sequence is reasonable (19). MR estimates the causal relationship between AFS, AFB, and PPD using single nucleotide polymorphisms (SNPs) as instrumental variables (IVs) (20).

## 2 Materials and methods

**Figure 1:**
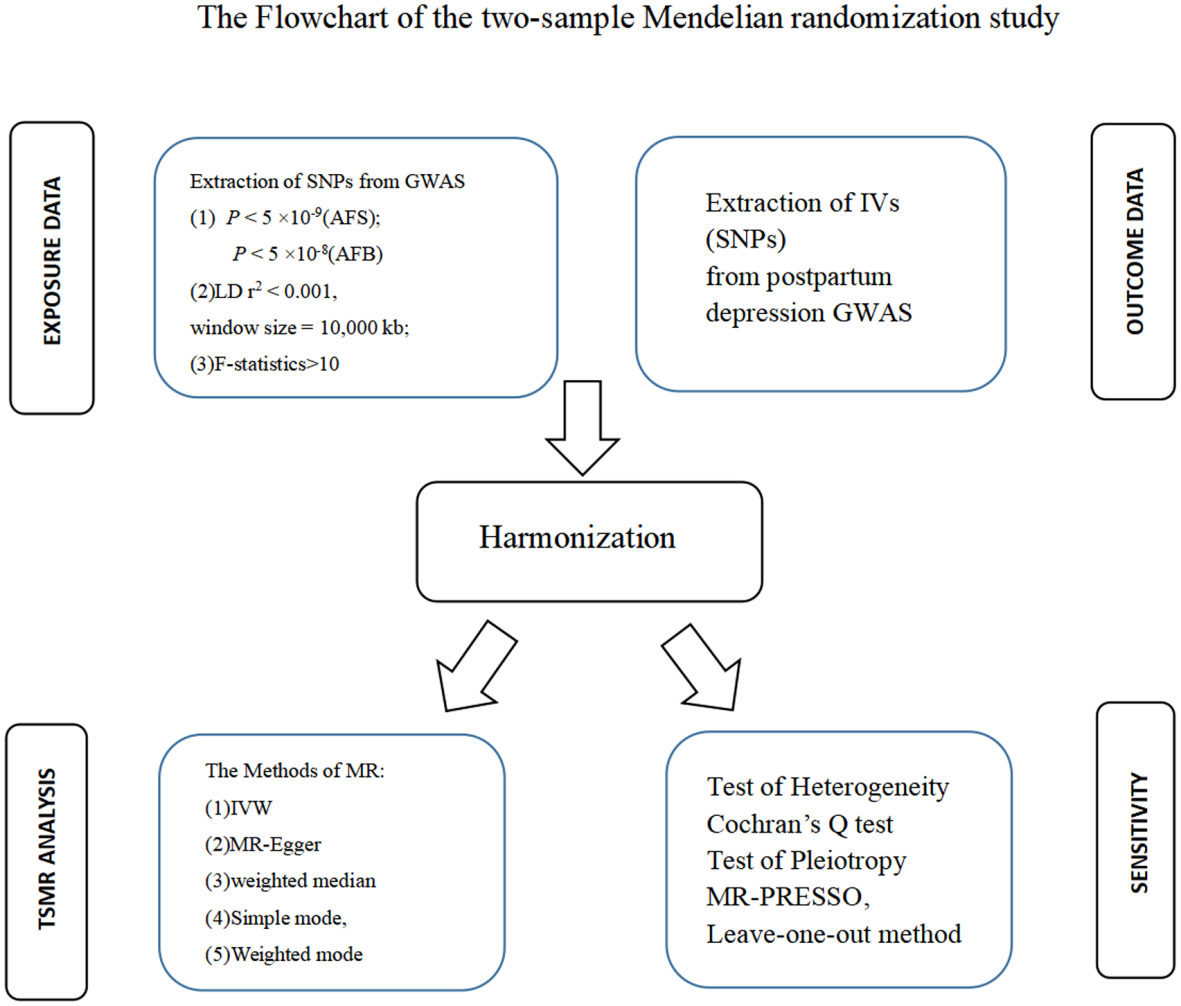
Flowchart of the two-sample Mendelian randomization study.

Analysis of our MR (the flowchart is shown in **Figure 1**) was conducted to determine causal relationships between exposures and outcomes. The summary data for the relationships between AFS or AFB and PPD were extracted from GWAS summary datasets. AFS-related SNP data included 406,457 individuals of European ancestry (ukb-b-6591). AFB-related SNP data included 170,498 individuals of European ancestry (ukb-b-12405). PPD-related SNP data comprised 7,604 patients and 59,601 controls of European ancestry (finn-b-O15_POSTPART_DEPR).

### 2.1 Selection of instrumental variables (IVs)

Three basic assumptions are prerequisites for completing MR: (1) IVs must be significantly correlated with the exposure. Second, IVs should influence the risk of outcomes only via exposure. Third, IVs are unrelated to any confounding factors, which are associated with exposure and the risk of outcome.

We selected the IVs according to the *P* value of exposure-related SNPs (*P*<5 ×10^-9^ for AFS-related SNP and *P*<5 ×10^-8^ for AFB-related SNP^)^ and estimated the linkage disequilibrium (LD) using the clump parameter (10,000 kb, LD r^2^ < 0.001). SNPs were considered IVs that were significantly associated with exposure factors without LDs. The F-statistics of the IVs should be > 10, supporting that the IVs confidently predict exposure (21, 22). The IVs were not directly related to the risk of outcome (*P*>5 ×10^-8^).

### 2.2 Statistical analysis

We performed a statistical analysis (R version 4.2.2 software) for two-sample MR (TSMR) using the “TwoSampleMR” and “MR-PRESSO” packages. We primarily assessed the causality between AFS and the risk of PPD using a random-effects model of IVW (the main method for MR), the method of weighted median, the method of MR−Egger, the simple mode method and the weighted mode method. Underlying pleiotropy should be necessarily tested to confirm that genetic IVs are unrelated to all confounding factors associated with both AFS and the risk of PPD. Cochran’s Q test was used to detect heterogeneity between the IVs (IVW and MR Egger methods), and a value of *P* less than 0.05 indicated the presence of heterogeneity (POH). A nonzero intercept derived from MR−Egger regression was considered POH (*P* < 0.05). MR-PRESSO, which is composed of three components (global testing, outlier testing, and distortion testing), was used to detect the presence of pleiotropy (*P* < 0.05). The method of MR-Egger should be used as the main method for TSMR analyses in the presence of pleiotropy (23). The leave-one-out method should also be used to assess the impact of a single IV on the TSMR estimate. *P* < 0.05 was considered evidence of pleiotropy. Then, we assessed the causality between AFB and the risk of PPD using the same method described above.

## 3 Results

### 3.1 The causal relationship between AFS and PPD

#### 3.1.1 TSMR analysis

**Figure 2:**
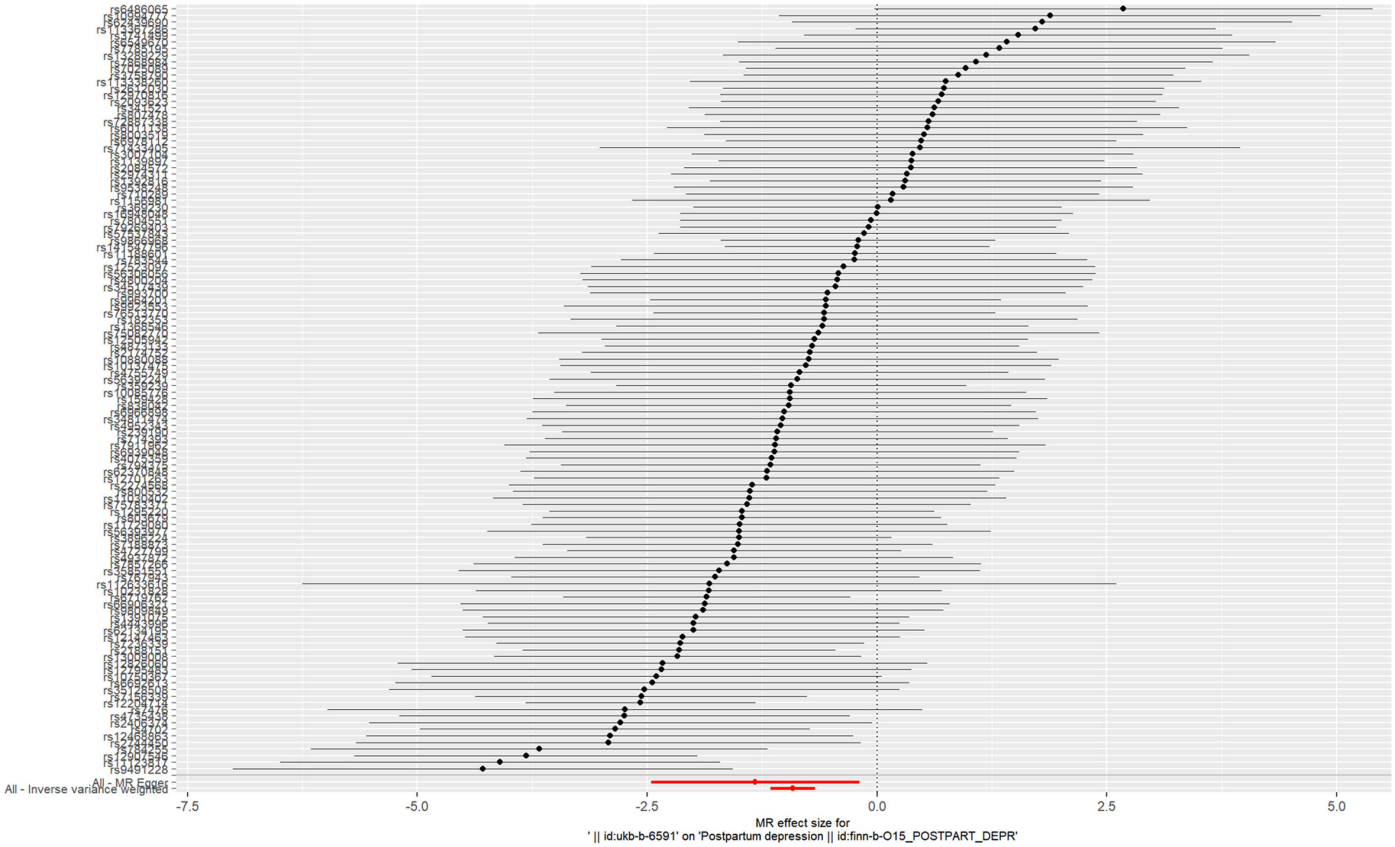
Forest plot of age at first sexual intercourse associated with postpartum depression.

**Figure 3:**
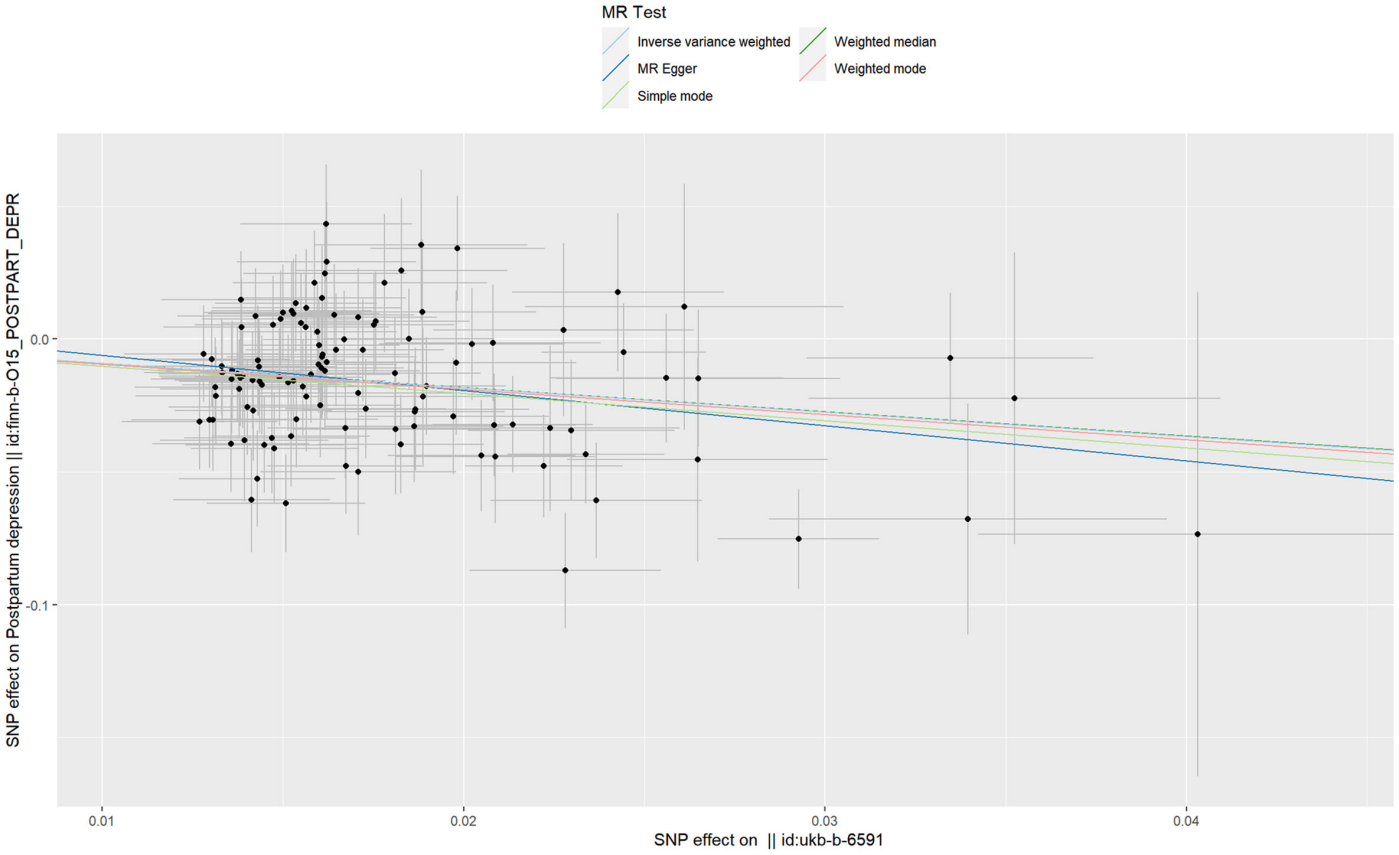
Scatter plot of age at first sexual intercourse associated with postpartum depression.

The IVs of AFS were the 116 SNPs with F-statistics >10. The causal effect between AFS and the risk of PPD was revealed by the results of our TSMR as following odds ratios: (1) odds ratio = 0.4, *P <*0.001 (IVW); (2) odds ratio = 0.27, *P* =0.024 (MR-Egger); (3) odds ratio = 0.4, *P <*0.001 (weighted median); (4) odds ratio = 0.36, *P* > 0.05 (simple mode); and (5) odds ratio = 0.39, *P*> 0.05 (weighted mode). Significant differences were found using the three methods (MR-egger, IVW, and weighted median) (**Figure 2**). The results of all the five methods (ORs) are all less than 1. These results indicate that as AFS increases, the risk of PPD decreases. Therefore, we believe that early AFS leads to an increased risk of PPD (**Figure 3**).

#### 3.1.2 Sensitivity and pleiotropy analyses

**Figure 4:**
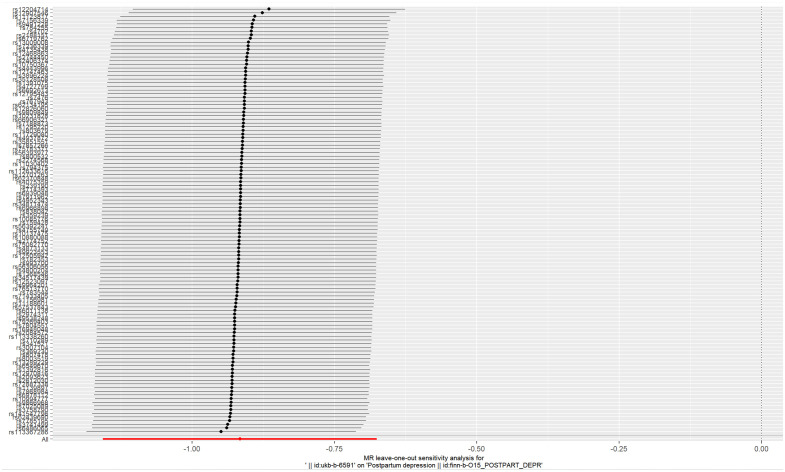
Plot of leave-one-out sensitivity analysis (IVW).

Cochransp Q-test showed values of *P* less than 0.05 (0.044 for MR Egger and 0.047 for IVW), indicating that the heterogeneity among the 116 AFS-related SNPs was significant. The MR-PRESSO test confirmed that no significant pleiotropy was detected (*P*=0.058). Significant pleiotropy was also not found according to the intercept (*P=*0.472), which was obtained from the MR−Egger regression. Sensitivity analyses (leave-one-out method) were also performed. Moreover, the pooled estimate (beta) of the TSMR cannot be fundamentally impacted by each of the 116 SNPs (**Figure 4**). The results derived from the random-effects model of IVW about the causal relationship between AFS and PPD are robust.

### 3.2 The causal relationship between AFB and PPD

#### 3.2.1 TSMR analysis

**Figure 5:**
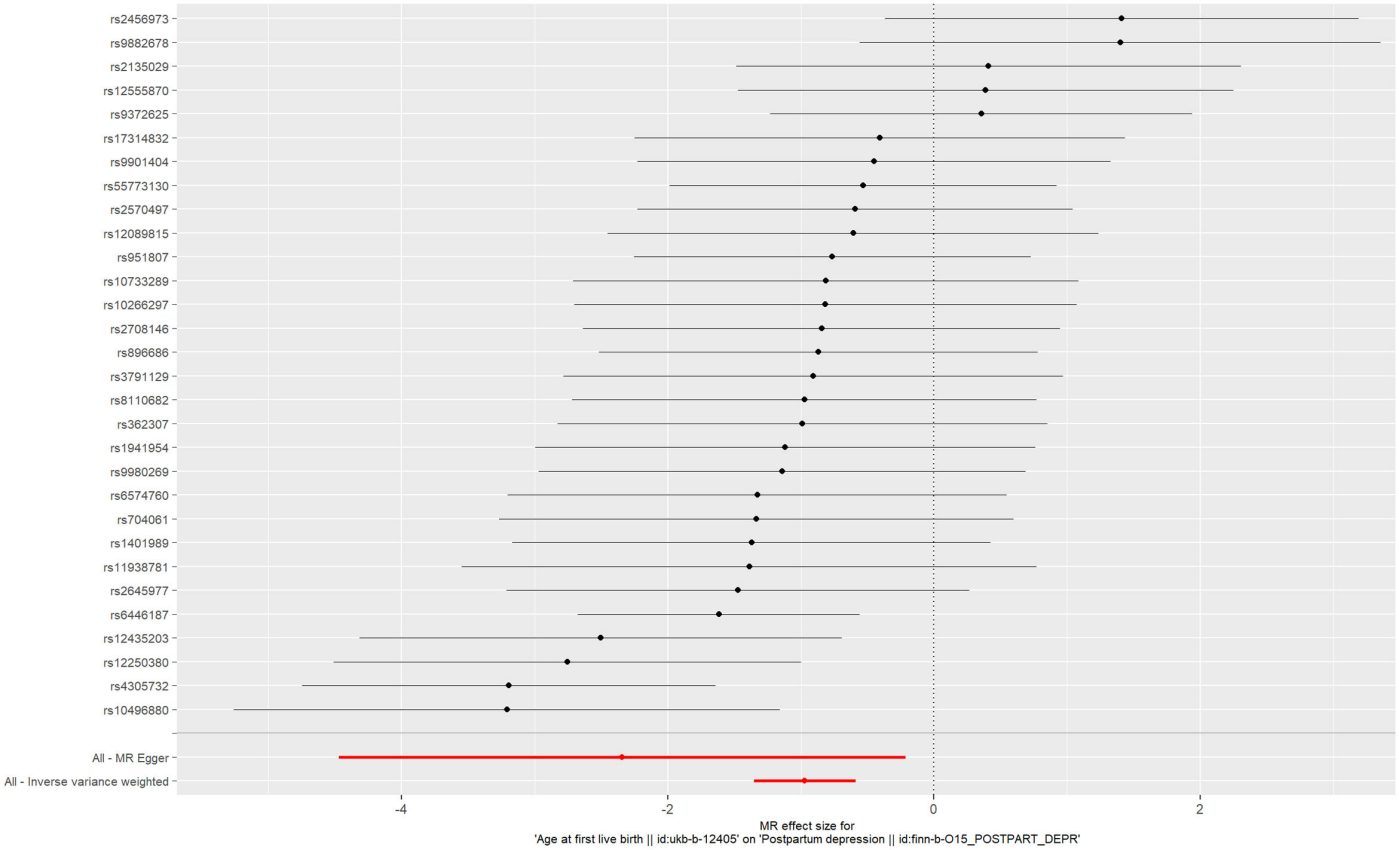
Forest plot of age at first live birth associated with postpartum depression.

**Figure 6:**
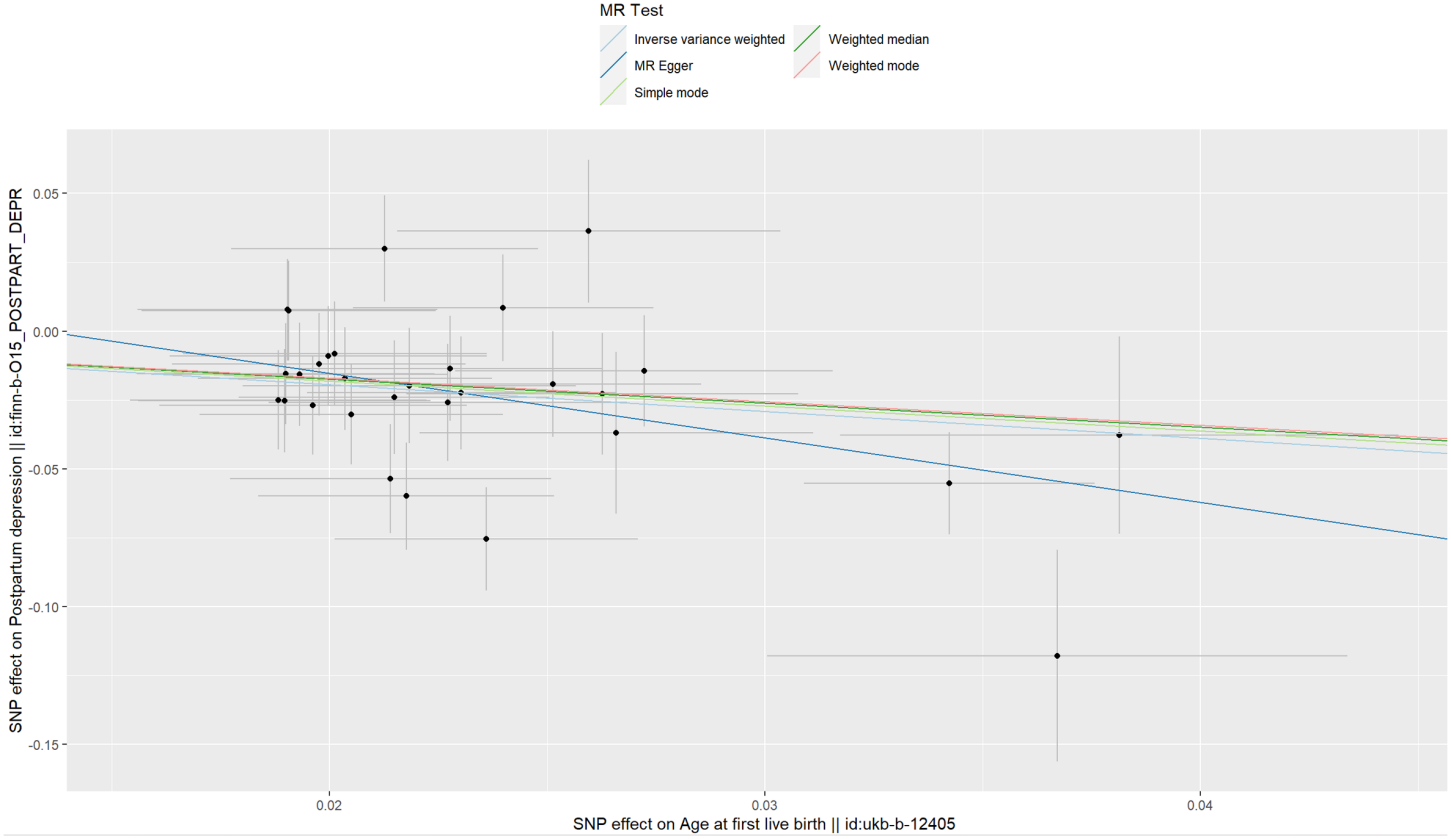
Scatter plot of age at first live birth associated with postpartum depression.

The IVs of AFB were the 30 SNPs with F-statistics >10. The causal effect between AFB and the risk of PPD was revealed by the results of our TSMR as following odds ratios: (1) odds ratio = 0.38, *P <*0.001 (IVW); (2) odds ratio = 0.10, *P* = 0.04 (MR-Egger); (3) odds ratio = 0.42, *P <*0.001 (weighted median); (4) odds ratio = 0.40, *P* > 0.05 (simple mode); and (5) odds ratio = 0.43, P > 0.05 (weighted mode). Significant differences were found using the main methods (IVW) and the other two methods (MR-Egger, weighted median) (**Figure 5**), and the ORs of all the five methods were less than 1. These results indicate that as AFB increases, the risk of PPD decreases. Therefore, we believe that early AFB leads to an increased risk of PPD (**Figure 6**).

#### 3.2.2 Sensitivity and pleiotropy analyses

**Figure 7:**
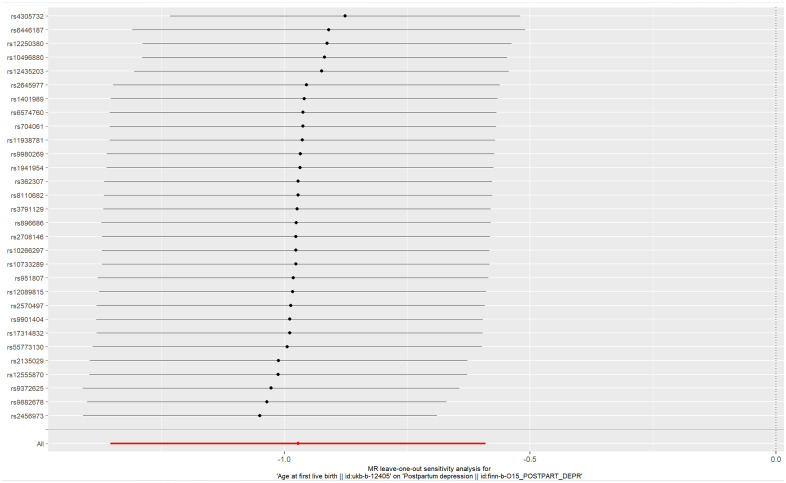
Plot of leave-one-out sensitivity analysis (IVW).

Cochran’s Q-test showed values of *P* above 0.05 (0.065 for MR Egger and 0.051 for IVW), indicating that the heterogeneity between the 30 genetic variants of AFB was insignificant. The MR-PRESSO test confirmed that no significant pleiotropy was detected (*P=*0.066). Significant pleiotropy was also not found according to the intercept (*P=*0.211), which was obtained from the MR−Egger regression. Sensitivity analyses (leave-one-out method) were also performed. Moreover, the pooled estimate (beta) of the TSMR could not be fundamentally impacted by each of the 30 SNPs (**Figure 7**). The 30 genetic variants (SNPs) can be used as IVs for the analysis (IVW method) of TSMR. The results of our TSMR about the causal relationship between AFB and PPD are robust.

## 4 Discussion

Previous MR studies have shown that both early AFS and early AFB are risk factors for major depressive disorder (MDD) (24, 25). Most studies, including control groups of nonperinatal women, have concluded that PPD differs from nonperinatal depression in women (26, 27). There is currently no consensus about a significant clinical debate as to whether PPD differs enough from MDD to warrant a separate diagnosis, but treating early-onset PPD as a disorder different from MDD is more advantageous (28–31). PPD may present complex phenotypes of several diseases via different pathways (29). In the postpartum period, women with PPD are significantly more likely to exhibit bipolar symptoms, and bipolar PPD has a significantly earlier age of onset than unipolar depression (7, 8, 32). It is necessary to distinguish bipolar PPD from unipolar depression to help us understand the conflicting outcomes (33). Epidemiological studies have shown a negative correlation between maternal depression symptoms and maternal age (34, 35). However, another study suggested that women aged 32 and above have an increased risk of psychological distress compared to women aged 25-31 (36). Some studies also suggest that AFB is not related to PPD or exhibits a nonlinear relationship (13, 14). The exploration of causal relationships in epidemiological studies is influenced by confounding factors, such as familial confounding (37). Due to the unclear causal relationships between AFS or AFB and PPD, it is necessary to conduct a TSMR study on this controversial topic.

The causal inference between AFS, AFB, and PPD was evaluated by our TSMR study. When there was no pleiotropy, the TSMR was completed using the method of IVW (a random-effects model) as the main method (38, 39). When pleiotropy existed, we mainly used the method of MR-Egger (23). We found that both early AFS and early AFB are risk factors for PPD, and preventing early AFS is an effective way to delay early AFB. Girls who start sexual intercourse in early adolescence are more likely to conceive and have children during adolescence than girls who only start sexual intercourse during adolescence age (40). Therefore, early AFS is a more important risk factor that needs to be taken seriously. Sexual education for young girls may reduce the negative consequences of early sexual behavior (41).

There are several strengths in our study: (1) In the absence of sufficient epidemiological studies and consistent conclusions, we completed the first MR study on this issue. The results of the TSMR provides new evidence for us to understand the roles of AFS and AFB in PPD. (2) Large-scale GWAS datasets were used for our studies (397338 individuals of European ancestry for AFB and AFS, and 67205 individuals of European ancestry for PPD). (3) To obtain robust results, five computational methods (the random effects model of IVW, the method of weighted median method, the simple mode method, the method of MR−Egger, and the method of weighted mode) were used. However, several limitations should be discussed in our TMSR. (1) The results of our TSMR may not be suitable for non-European populations and should be verified in various races in the future. (2) Horizontal pleiotropy cannot be absolutely eliminated, and the results of our TSMR should be verified by high-quality prospective epidemiological studies. (3) The TSMR failed to explore the mechanism through which AFS and AFB affect PPD.

## 5 Conclusion

The MR study based on GWAS data revealed that both AFS (OR= 0.4, *P <*0.001) and AFB (OR= 0.38, *P <*0.001) were negatively correlated with the risk of PPD. Early AFS and early AFB should be studied as possible risk factors for PPD in the future. Public health departments should attach importance to sex education for young girls. The results of our TSMR should be verified by high-quality prospective epidemiological studies in the future.

## Data availability statement

Publicly available datasets were analyzed in this study. This data can be found here: GWAS summary data (https://gwas.mrcieu.ac.uk/).

## Author contributions

XZ: Writing – original draft, Writing – review & editing. LL: Writing – review & editing.
